# Enhancing anticancer peptide discovery: A fusion-centric framework with conditional diffusion for prediction and generation

**DOI:** 10.1371/journal.pcbi.1014098

**Published:** 2026-03-26

**Authors:** Binyu Li, Xin Zhang, Zhihua Huang, Prayag Tiwari, Quan Zou, Yijie Ding, Xiaoyi Guo

**Affiliations:** 1 School of Computer Science and Technology, Xinjiang University, Urumqi, China; 2 Yangtze Delta Region Institute (Quzhou), University of Electronic Science and Technology of China, Quzhou, China; 3 Hangzhou Institute of Medicine, Chinese Academy of Sciences, Hangzhou, China; 4 School of Information Technology, Halmstad University, Halmstad, Sweden; 5 Faculty of Computing, Harbin Institute of Technology, Harbin, China; 6 Li Ka Shing Faculty of Medicine, The University of Hong Kong, Hong Kong SAR, China; Georgia Institute of Technology, UNITED STATES OF AMERICA

## Abstract

Anticancer peptides (ACPs) are short bioactive sequences that selectively target tumor cells with minimal toxicity, positioning them as promising candidates for next-generation cancer therapies. However, existing computational models face limitations in sequence representation and class imbalance. To address these challenges, we propose UACD-ACPs, a unified fusion-driven framework that integrates a diffusion-inspired noise-conditioned classifier for ACP prediction and a diffusion-based peptide generation module with cancer-type-aware organization for targeted downstream screening. The classification module integrates ProtBERT-based semantic embeddings with physicochemical descriptors via the Multiscale Embedding Compression Strategy (MECS) and a diffusion-inspired noise-conditioned encoder, substantially enhancing predictive robustness and accuracy, particularly under challenging imbalanced multi-class settings. In the generative pipeline, we introduce a denoising diffusion-based generative framework augmented by two novel fusion modules: the Bitemporal Fusion Module (BFM) and the Temporal Feature Attention Module (TFAM). These modules perform multi-scale temporal and semantic fusion to promote the generation of structurally coherent and functionally relevant peptide candidates. Experimental results demonstrate that UACD-ACPs outperforms state-of-the-art methods in terms of accuracy, F1-score, and AUC-ROC. The generated peptides exhibit favorable physicochemical properties, diverse secondary structures, and strong structural stability, as validated by molecular dynamics simulations and membrane-binding analyses. Overall, this study highlights the potential of fusion-driven diffusion-based frameworks for alleviating class imbalance and data heterogeneity in anticancer peptide modeling, paving the way for scalable and biologically grounded ACP discovery.

## Author summary

Anticancer peptides (ACPs) are short protein sequences that can selectively target tumor cells while causing minimal harm to healthy tissues, making them promising candidates for cancer therapy. However, the computational discovery of ACPs remains challenging because peptide sequences are highly diverse and data across different cancer types are often severely imbalanced. In this study, we propose UACD-ACPs, a unified computational framework designed to support both the prediction and generation of anticancer peptides. By integrating complementary sequence representations with physicochemical information, the framework improves the reliability of ACP identification under challenging imbalanced data conditions. In addition, the model enables the generation of novel peptide candidates organized by cancer type, facilitating targeted downstream screening. Experimental results demonstrate that UACD-ACPs achieves improved predictive performance compared with existing methods. The generated peptides also exhibit favorable physicochemical properties and structural stability, suggesting their potential biological relevance. Overall, this work provides an integrated and scalable computational strategy to support anticancer peptide discovery and guide future experimental studies.

## 1 Introduction

Cancer remains one of the leading threats to global public health, causing millions of deaths each year [1]. Anticancer peptides (ACPs) are short peptides capable of selectively recognizing and killing cancer cells. Due to their high target specificity and low toxicity, ACPs have emerged as promising therapeutic candidates [2–4]. With the rapid progress of peptide-based drug research, ACPs are increasingly regarded as effective complements to conventional therapies [5]. A key advantage of ACPs lies in their ability to selectively target malignant cells while sparing healthy tissues [6,7]. Their mechanisms of action include both membrane-dependent and membrane-independent pathways, such as membrane disruption, apoptosis induction, inhibition of angiogenesis, immune modulation, DNA damage, and suppression of cell proliferation [8]. These unique features support their potential as a new class of anticancer agents. Despite their promise, ACPs are still far from clinical application. Most investigations remain confined to *in vitro* or early-stage trials, with progress hindered by enzymatic instability, poor bioavailability, and insufficient tumor-targeting efficiency [9,10]. To overcome these challenges, diverse strategies have been proposed, including chemical modification of peptides, nanocarrier-based delivery systems, and computational design approaches [11,12]. Nevertheless, the intrinsic short length and functional diversity of ACP sequences make it difficult to accurately extract functional features directly from amino acid sequences [13]. Recent advances in deep learning and protein language-model embeddings have opened new avenues for peptide sequence representation and activity prediction, by enabling the automatic extraction of rich contextual, semantic, and evolutionary information directly from raw amino acid sequences, thereby reducing reliance on manually engineered features [14–16]. However, most existing approaches primarily focus on peptide activity prediction, while failing to jointly consider sequence generation, structural stability, and downstream biophysical validation in an integrated framework.

Building on these developments, we propose a conditional diffusion-based framework for Anticancer peptide (ACP) prediction and generation. Our method integrates multi-level evaluation, molecular docking, and simulation analyses to simultaneously address peptide stability, bioactivity, and structural compatibility. This framework aims to accelerate rational ACP design and facilitate their clinical translation.

In recent years, multiple computational tools have been developed to support ACP identification and design. Wang et al. introduced SBSM-Pro, a bio-sequence machine learning tool that improves ACP prediction accuracy [17]. He et al. developed MRMD3.0, which integrates dimensionality reduction and visualization strategies to facilitate ACP analysis [18]. Guo et al. proposed a recognition method based on sequence homology scoring using a deep fuzzy network, enhancing prediction accuracy and offering new perspectives for peptide design [19]. These methods provide effective technical support for ACP prediction and have accelerated progress in the field.

In ACP identification, traditional machine learning methods still play an important role. These approaches rely on manually extracted physicochemical features such as amino acid composition, hydrophobicity, and isoelectric point, combined with classifiers including support vector machines (SVMs) and random forests (RFs) [20]. ACPScanner integrates SVM, RF, and XGBoost with 25 physicochemical descriptors, achieving 92% accuracy across multiple datasets [21]. MLACP improves modeling of complex peptides through optimized feature selection [22]. StackACPred applies multi-feature fusion and stacked ensembles, reaching an accuracy of 86% [23]. ACP_MS combines amino acid composition with dipeptide frequency to improve performance on benchmark datasets [24]. EnACP employs spectral features (e.g., PSSM-DT, PSSM-CC) with an LGBM classifier, enhancing efficiency and stability [25]. Although these models perform well on small to medium-sized datasets, they depend heavily on manual feature construction, making it difficult to capture complex semantic and structural patterns in sequences. Consequently, their generalizability remains limited [26].

In recent years, deep learning has been increasingly applied to ACP identification due to its end-to-end feature extraction capabilities. In contrast, DeepACP adopts a recurrent neural network (RNN) architecture to capture sequential dependencies within peptide chains [27]. Focusing on interpretability, ACP-MLC integrates a hierarchical prediction strategy with the SHAP algorithm to elucidate model decision processes [28]. ACP-DL combines long short-term memory (LSTM) networks with binary spectrum profiles and k-mer sparse matrices to construct expressive embeddings [29], whereas ACP-MCAM incorporates multicore CNNs with attention mechanisms, reaching 88% accuracy [30]. ACPNet fuses physicochemical descriptors with sequence embeddings and integrates RNNs and fully connected layers [31]. ACP-MHCNN uses multimodal inputs and multihead convolutional structures to enhance representation capacity [32], while DLFF-ACP applies a dual-channel parallel architecture to separately capture spatial and contextual features [33]. Finally, ANNprob-ACPs integrates nine feature encoding methods and multiple submodels into a meta-learning framework to improve predictive robustness [34]. Despite these advances, deep learning models still face challenges such as overfitting, limited interpretability, and unstable performance under small sample sizes, class imbalance, and high functional diversity [35,36]. In parallel, recent studies have increasingly explored hybrid, multimodal, and explainable learning paradigms to enhance peptide representation and functional prediction across diverse biological tasks. Hybrid frameworks that integrate protein language model embeddings with conventional physicochemical descriptors have demonstrated improved predictive performance and interpretability, as exemplified by HyPepTox-Fuse for peptide toxicity prediction [37] and xBitterT5, an explainable transformer-based multimodal architecture for bitter-taste peptide identification [38]. In the context of anticancer peptides, mACPpred 2.0 adopts a stacked deep learning strategy that combines spatial and probabilistic feature representations to improve the robustness of ACP identification [39]. Beyond ACP prediction, advanced modeling strategies have also been extended to related peptide–biomolecule interaction problems, including the multi-view learning framework based on Taylor expansion theory in CFCN for HLA–peptide binding prediction [40] and the feature-optimized machine learning approach in APLpred for asparagine peptide lyase characterization [41]. Moreover, transformer-based architectures have demonstrated strong generalizability in broader biological contexts, such as MST-m6A for multi-scale prediction of m^6^A RNA modification sites across diverse cellular environments [42]. Complementarily, multimodal and explainable graph-based frameworks such as XMolCap further highlight the potential of integrating graph neural networks with interpretability mechanisms for molecular understanding and generation [43].

Researchers have expanded modeling tasks to include not only ACP prediction but also design and generation [44]. Current sequence generation approaches such as generative adversarial networks (GANs) [45], variational autoencoders (VAEs) [46], and flow-based frameworks [47] have shown promise in improving sequence modeling. However, GANs often suffer from training instability and mode collapse, especially under small-sample conditions, limiting their ability to produce diverse and effective peptides. Similarly, VAEs can capture spatial representations, but the peptides generated often lack local fidelity and structural consistency. Furthermore, complex deep models, due to their intricate architectures, redundant parameters, and high computational cost, remain impractical for large-scale applications in biological sequence analysis.

Therapeutic peptide research faces several challenges: (1) severe class imbalance in ACP datasets limits model training and accuracy; (2) high sequence diversity and structural complexity hinder effective feature extraction; (3) deep models trained on small, imbalanced data are prone to overfitting and instability.

To address these issues, we propose UACD-ACPs, a unified fusion-driven framework that leverages diffusion-inspired noise conditioning for ACP classification and an attention-enhanced diffusion-based generative module with cancer-type-aware organization for peptide generation. (1) SMOTE-based oversampling [48] is employed for minority-class synthesis, while a diffusion-inspired noise-conditioned encoder integrates semantic label information to improve robustness under class-imbalanced settings; (2) the MECS module incorporates multiscale global–local attention to enhance representation learning and interpretability; (3) for peptide generation, a denoising diffusion-based generative framework augmented with the Bitemporal Fusion Module (BFM) and the Temporal Feature Attention Module (TFAM) promotes the synthesis of structurally stable and functionally relevant peptide candidates, which are subsequently organized in a cancer-type-aware manner for downstream screening and analysis. Generated sequences are further evaluated through molecular dynamics simulations and biological analysis.

In addition to extensive preclinical investigations, several anticancer peptide–based agents have progressed into early-stage clinical evaluation, underscoring the translational potential of ACPs. One representative example is p28, a peptide derived from the bacterial protein azurin, which exhibits tumor-suppressive activity by stabilizing p53 and disrupting oncogenic protein interactions; early-phase clinical studies have primarily focused on evaluating its safety and preliminary antitumor efficacy in patients with solid tumors [49]. Another peptide-derived therapeutic, sulanemadlin (ALRN-6924), is a stapled *α*-helical peptide designed to reactivate p53 through inhibition of MDM2/MDMX interactions and has entered Phase I clinical trials for hematologic malignancies and selected solid tumors, although dose-limiting toxicities have been reported in some studies [50]. In addition, pegdinetanib (CT-322), a VEGFR-2–targeting peptide monobody, advanced to Phase II clinical trials for glioblastoma and other solid tumors, demonstrating the feasibility of peptide-based inhibition of tumor angiogenesis in clinical settings [51]. Collectively, these examples indicate that ACPs and peptide-based biologics are not merely theoretical candidates but are actively being explored in early-stage clinical contexts, further highlighting the need for robust and efficient computational frameworks to accelerate rational ACP design and optimization.

**Fig 1:**
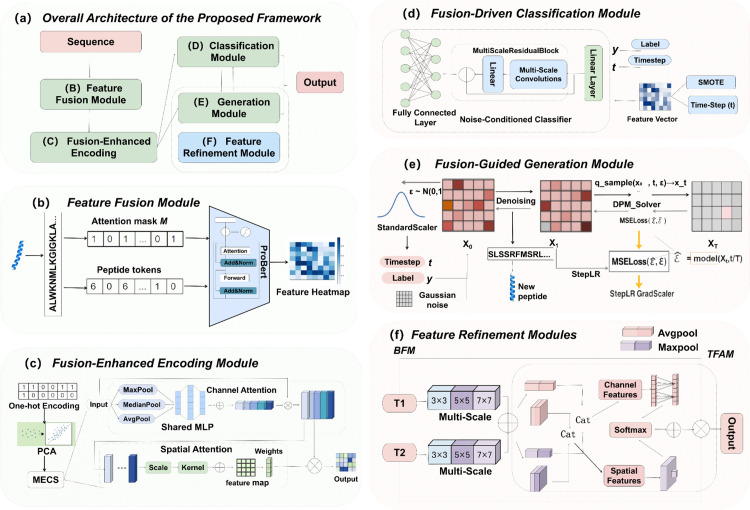
Overall architecture of the proposed fusion-driven framework for anticancer peptide classification and generation. **(a)** Overall Architecture: The framework takes peptide sequences as input and processes them through five interconnected modules, including Feature Fusion, Fusion-Enhanced Encoding, Classification, Generation, and Feature Refinement, to produce predictive and generative outputs. **(b)** Feature Fusion Module: Converts peptide sequences into token embeddings and attention masks using a pretrained ProtBERT model, generating semantically enriched feature representations. **(c)** Fusion-Enhanced Encoding Module: Integrates physicochemical features and semantic embeddings via multiscale convolutions and spatial–channel attention mechanisms. One-hot encoding and PCA are applied for dimensionality alignment. **(d)** Fusion-Driven Classification Module: A diffusion-inspired noise-conditioned classifier with multiscale residual blocks predicts anticancer peptide classes under imbalanced data settings, leveraging SMOTE and stochastic time-step conditioning. **(e)** Fusion-Guided Generation Module: Generates peptide representations from noise using a diffusion-based generative process, followed by cancer-type-aware organization **(f)** Feature Refinement Modules: Two modules, BFM (Bitemporal Fusion Module) and TFAM (Temporal Feature Attention Module), integrate multiscale temporal and spatial features through one-dimensional and two-dimensional convolutions to enhance the biological fidelity of generated peptide candidates.

As shown in Fig 1, UACD-ACPs unifies classification and generation within a single architecture, addressing key limitations in ACP discovery through deep feature fusion and biologically grounded validation.

## 2 Methods

This study proposes UACD-ACPs, a unified framework for ACP classification and cancer-type-aware peptide generation. For classification, ProtBERT embeddings [52] and physicochemical features are fused and refined using the multiscale enhanced convolutional structure (MECS) and a diffusion-inspired noise-conditioned encoder. The classification module and generation module share a common feature space, meaning both modules process the same set of features extracted from the dataset. While they share this feature space, each module operates independently during training, learning different representations based on their respective tasks. This shared feature space enables information exchange between the modules and allows them to complement each other’s learning processes. For peptide generation, a denoising diffusion-based generative framework incorporating the Bitemporal Fusion Module (BFM) and the Temporal Feature Attention Module (TFAM) is employed to promote structural coherence and functional relevance of the generated peptide candidates. These modules are trained separately, with their learning processes aligned through their shared feature space, optimizing both the classification and generation tasks. The generated peptides, ranging from 8 to 50 amino acids in length, are further evaluated through physicochemical analysis, AlphaFold2-based structure modeling, and molecular dynamics simulations, supporting their biological plausibility [53]. Code and data are available at https://github.com/yidingneng/ACP-ConditionalDiffusion.git.

### 2.1 Multiscale fusion feature extraction module

To enhance the classification accuracy and representation capacity for anticancer peptides (ACPs), we propose a multiscale feature extraction module that integrates pretrained semantic embeddings with sequence-derived physicochemical descriptors, as illustrated in Fig 1b. Semantic representations are obtained using ProtBERT, a protein-specific language model trained via self-supervised learning on large-scale protein sequence corpora.

Each peptide sequence is encoded as a 1024-dimensional embedding, corresponding to the native hidden representation size of ProtBERT, which captures contextual dependencies and latent structural semantics within the amino acid sequence.

Compared with traditional handcrafted features, ProtBERT embeddings provide improved generalizability and richer context-aware representations, enabling more effective modeling of peptide functionality.

In parallel, a 430-dimensional vector of physicochemical descriptors is extracted for each peptide, including amino acid composition, dipeptide frequency, isoelectric point, and hydrophobicity-related properties.

These physicochemical features offer complementary biochemical and structural information that supports model interpretability and enhances discriminative capability. Notably, the resulting feature dimensionality is determined by predefined encoding schemes rather than manual parameter tuning.

By concatenating semantic embeddings with physicochemical descriptors, the proposed framework constructs a multiscale input representation, which improves robustness under data-limited conditions and facilitates the identification of functionally relevant ACPs.

### 2.2 MECS module

As shown in Fig 1c, the proposed MECS module enhances multiscale feature representation by sequentially applying channel and spatial attention mechanisms, thereby improving the model’s ability to recognize complex anticancer peptide patterns. Unlike conventional backbone-level multi-scale designs, MECS achieves multi-scale feature compression and fusion within the embedding space by jointly modeling channel-wise statistical variations and spatial dependencies at different receptive fields, thereby reducing feature redundancy while preserving both global semantic information and local fine-grained details.

The channel attention submodule captures global semantic importance by applying max, average, and median pooling across channels to extract statistical descriptors. These descriptors are passed through a shared multilayer perceptron (MLP) to generate channel-wise importance weights, which are projected back to the original feature maps to emphasize discriminative channels and retain biologically relevant information.

To model local dependencies, the spatial attention submodule performs multiscale convolutions on the input features. Pointwise convolution is used to compute two-dimensional spatial attention weights, which highlight functionally significant sequence regions and enhance local resolution.

The outputs from both attention branches are fused to generate features that are both channel-selective and spatially aware. This integration strengthens interscale correlations while preserving semantic consistency across feature hierarchies.

The MECS computation consists of three main stages: global perception, channel attention, and spatial attention. First, global perception is applied to the input feature **X** via a 1×1 convolution followed by a GELU activation, as defined in Equation (1), producing an intermediate representation **F** (Equation (2)). Here, *W*_*p*_ denotes the weight matrix of the first pointwise convolution used for global channel transformation.


$$𝐗\in {\mathbb{R}}^{B\times C\times H\times W}$$ (1)

$$X^{\prime }=\text{GELU}(W_{p}*𝐗)$$ (2)

Next, the channel attention mechanism aggregates global information by applying average, max, and median pooling across the spatial dimensions of the feature maps, producing three channel descriptors. Each descriptor is passed through a shared two-layer 1×1 convolutional network (Conv-ReLU-Conv), functionally equivalent to a multilayer perceptron, to capture nonlinear dependencies across channels. The resulting outputs are activated using the Sigmoid function to produce three channel-wise attention maps: *A*_avg_, *A*_max_, and *A*_med_. These are summed to obtain the final channel attention map *A*_*c*_, as defined in Equation (3):


$$A_{c}=A_{\text{avg}}+A_{\text{max}}+A_{\text{med}}$$ (3)

The attention map is then multiplied with the input feature on a channel-wise basis to achieve adaptive enhancement along the channel dimension, resulting in the adaptive feature ${\text{X}}_{\text{c}}$, as shown in Equation (4).


$$X_{c}=A_{c}⊙X^{\prime }$$ (4)

Next, the process proceeds to the spatial attention module. A 5×5 depthwise separable convolution is first applied for initial spatial modeling. To capture long-range directional dependencies, multiple depthwise convolutions with asymmetric kernels (1×7, 7×1, 1×11, 11×1, 1×21, 21×1) are used. The outputs of these convolutions are summed and fused to produce the spatially enhanced representation ${\text{X}}_{\text{s}}$, as defined in Equation (5).


$$X_{s}=\sum_{i=1}^{n}{\text{DepthConv}}_{k}({\text{DepthConv}}_{k}(X_{c}))$$ (5)

The fused features are passed through a 1×1 convolution to generate the spatial attention map, which is element-wise multiplied with the channel-enhanced features. Subsequently, a final 1×1 convolution is applied to integrate the combined attention features, producing the output representation $X_{out}$, as shown in Equation (6). Here, *W*_*p*_’ denotes the weights of the spatial attention convolution, and *W*_*p*_” represents the weights of the final pointwise convolution used to project the fused features into the output space.


$$X_{out}=W_{p}^{\prime }*\left[({W_{p}^{\prime }}^{\prime }*X_{s})⊙X_{c}\right]$$ (6)

In summary, the MECS module leverages global perception for initial feature refinement and fuses channel and spatial attention to capture multi-scale contextual information, significantly enhancing feature representations.

### 2.3 Fusion-driven predictive classification module

An efficient ACP classification model was developed by integrating multiscale residual structures, a diffusion-inspired noise-conditioning module, and data augmentation techniques to effectively capture complex sequence patterns and improve prediction accuracy, as shown in Fig 1d.

During preprocessing, peptide sequences and labels are standardized and encoded. To address class imbalance, the Synthetic Minority Over-sampling Technique(SMOTE) oversampling is combined with a stochastic time-conditioning strategy, which perturbs feature representations in a controlled manner, enhances minority-class diversity, and improves model generalizability.

The core architecture employs multiscale residual blocks, where parallel convolutional branches *G*_*i*_(**x**) extract features at different receptive fields. Residual connections preserve semantic continuity, and the outputs are fused using learnable weights *W*_*i*_ to form a multiscale representation, as defined in Equation (7). This design captures both local and global sequence features, improving the model’s ability to handle structural heterogeneity.


$$𝐲=𝐅(𝐱)+\sum_{i=1}^{K}{𝐰}_{i}\;G_{i}(𝐱)$$ (7)

Training incorporates hierarchical K-fold cross-validation to maintain class distribution across folds, with early stopping used to prevent overfitting. Model performance is evaluated using accuracy, recall, F1-score, AUC-ROC, and confusion-matrix analysis. The best-performing parameters from the validation sets are retained for final inference.

Within the feature extraction pipeline, a noise-conditioned encoder inspired by diffusion models serves as the primary representation learner. For each minibatch, a stochastic time scalar is sampled from a uniform distribution and embedded as an additional conditioning signal. This time-dependent embedding is fused with peptide features and cancer-type label embeddings to form noise-conditioned representations, which are then fed into the multiscale residual network. Instead of explicitly performing iterative denoising as in full diffusion models, the stochastic time conditioning implicitly regularizes the feature space and encourages the encoder to learn smooth, robust decision surfaces.

This mechanism improves representation diversity and reduces overfitting, particularly for underrepresented cancer-type classes. By coupling SMOTE-based oversampling with noise-conditioned feature learning, the proposed model achieves a robust and generalizable solution for classifying variable-length, functionally diverse ACP sequences under imbalanced multi-class settings.

Algorithm 1 outlines the training procedure of the proposed ACP classifier with the biological objective of learning robust and cancer-type-specific representations of anticancer peptides. Each peptide is represented by fused ProtBERT embeddings and sequence-derived physicochemical descriptors, capturing both contextual sequence semantics and biologically relevant physicochemical properties associated with anticancer activity. To address the pronounced class imbalance inherent in ACP datasets, SMOTE is applied at the minibatch level to expand minority-class peptide representations while preserving their underlying feature distributions. A stochastic time scalar is introduced as an auxiliary conditioning variable to simulate diffusion-inspired perturbations in the peptide feature space, encouraging the model to learn smooth and noise-tolerant representations that reflect biological variability rather than overfitting to individual samples. This time-dependent signal is jointly embedded with peptide descriptors and cancer-type label information before being processed by the multi-scale classification network. Model parameters are optimized using a class-weighted cross-entropy loss, and validation performance is used for model selection.

**Algorithm 1** Noise-conditioned ACP classification from fused peptide representations

**Input:** Training set $\left(X,Y_{\text{acp}},Y_{\text{cancer}}\right)$, number of epochs *E*, learning rate *η*, class weights *w*

**Output:** Optimized model parameters *θ*

1: **for** epoch = 1 **to** *E* **do**

2:  Sample a minibatch $(x,y_{\text{acp}},y_{\text{cancer}})\sim D$   ▷*x* contains fused ProtBERT embeddings and physicochemical descriptors

3:  x←Standardize(x)   ▷Normalize scales of heterogeneous descriptors

4:   $y_{\text{acp}}\leftarrow \text{LabelEncode}(y_{\text{acp}})$

5:  $\left(x,y_{\text{acp}})\leftarrow \text{SMOTE}(x,y_{\text{acp}}\right)$   ▷Alleviate ACP class imbalance in training minibatches

6:  Sample stochastic time scalar t~U(0,1)   ▷Diffusion-inspired perturbation to reflect biological variability

7:   $x_{\text{proj}}\leftarrow \text{FeatureEncoder}(x)$

8:  $c_{\text{proj}}\leftarrow \text{ConditionEncoder}(y_{\text{cancer}})$   ▷Encode cancer-type functional context

9:   $x_{\text{cond}}\leftarrow x_{\text{proj}}+c_{\text{proj}}+\text{TimeEmbed}(t)$

10:   $\hat{y}\leftarrow f_{\theta }(x_{\text{cond}})$

11:   $L\leftarrow \text{WeightedCrossEntropy}(\hat{y},y_{\text{acp}};w)$

12:   $\theta \leftarrow \theta -\eta \nabla_{\theta }L$

13: **end for**

14: Evaluate on validation set and retain best *θ*

### 2.4 Fusion-guided peptide generation module

This study introduces a diffusion-based sequence generation model for cancer-type-aware synthesis of functional anticancer peptides [54]. As shown in Fig 1e, the model follows a standard diffusion paradigm, consisting of a forward noise injection phase and a reverse denoising process. Cancer-type labels are incorporated as auxiliary semantic information to organize and guide downstream screening of generated peptides toward biologically relevant regions of the feature space, enhancing functional relevance and specificity.

The denoising network adopts a residual backbone enhanced by two custom modules: BFM and the Temporal Feature Attention Module (TFAM), as illustrated in Fig 1f. The BFM uses parallel convolutions with varying receptive fields to capture both local and global contextual features across peptide fragments, enabling multi-level structural representation. TFAM, on the other hand, integrates channel-wise and spatial attention mechanisms to refine feature flow and highlight functionally critical sequence regions.

These two modules collaborate to improve temporal feature representation. BFM first captures short- and long-range dependencies by processing features across different scales. The output is then refined by TFAM, which emphasizes the most biologically relevant regions by adaptively weighting the temporal features through attention mechanisms. The combined output from both modules ensures that the model can capture both broad patterns and fine-grained details in peptide sequences.

TFAM integrates channel and spatial attention to refine feature flow and emphasize structurally critical regions. It processes inputs from two temporal states (e.g., T1 and T2), each through multi-scale convolutions, and fuses them via attention-guided interactions. This design improves local feature awareness and enhances biological plausibility, as shown in Equation (8), where *σ* denotes the ReLU activation.


$${\hat{T}}_{i}=\sum_{k\in \{3,5,7\}}\sigma \left({\text{Conv}}_{k}^{k}(T_{i})\right),\;i\in \{1,2\}$$ (8)

Global average pooling and max pooling are applied to the feature maps from the two temporal states to extract global channel descriptors. These descriptors are concatenated to form the joint channel representation $P_{\varepsilon }$, as defined in Equation (9). Two parallel 1D convolutional networks are then applied to $P_{\varepsilon }$ to generate the channel attention maps for each temporal branch, as described in Equation (10).


$$P_{\varepsilon }=\left[\text{Avg}({\hat{T}}_{1}),\text{Max}({\hat{T}}_{1}),\text{Avg}({\hat{T}}_{2}),\text{Max}({\hat{T}}_{2})\right]$$ (9)

$$\left[\alpha_{c}^{\left(1\right)},\alpha_{c}^{\left(2\right)}\right]=\text{Softmax}\left(\left[\text{Conv}1D(P_{\varepsilon }),\text{Conv}1D(P_{\varepsilon })\right]\right)$$ (10)

Spatial attention is computed by taking the mean and maximum values of the feature maps along the channel dimension to generate spatial descriptors, as defined in Equation (11). The concatenated descriptors are passed through two identical 2D convolutional layers to produce the spatial attention maps for each temporal branch, as described in Equation (12).


$$P_{s}=\left[\text{Mean}({\hat{T}}_{1}),\text{Max}({\hat{T}}_{1}),\text{Mean}({\hat{T}}_{2}),\text{Max}({\hat{T}}_{2})\right]$$ (11)

$$\left[\alpha_{s}^{\left(1\right)},\alpha_{s}^{\left(2\right)}\right]=\text{Softmax}\left(\left[\text{Conv}2D(P_{s}),\text{Conv}2D(P_{s})\right]\right)$$ (12)

The channel and spatial attention maps are combined through weighted fusion with a residual term added to enhance stability, as shown in Equation (13). The outputs from the two temporal branches are integrated as a weighted sum, as defined in Equation (14), to obtain the final fused feature representation *F*_fused_. This fused feature is then passed through a linear layer to produce the final denoising prediction output.


$$\beta_{i}=1+\alpha_{c}^{\left(i\right)}+\alpha_{s}^{\left(i\right)},\;i\in \{1,2\}$$ (13)

$$F_{\text{fused}}=\beta_{1}⊙{\hat{T}}_{1}+\beta_{2}⊙{\hat{T}}_{2}$$ (14)

During training, mean square error (MSE) is used as the loss function to minimize noise prediction error, improving fitting accuracy and suppressing residual noise. Mixed precision training is employed to reduce memory usage and accelerate convergence. For sequence generation, the DPM-Solver sampler is integrated to accelerate reverse diffusion. As an ODE-based solver, it adaptively adjusts step sizes, reducing sampling steps by 3–5× while maintaining output quality and sequence diversity. The denoised feature vectors are decoded into amino acid sequences of 8–50 residues, aligning with typical therapeutic peptide lengths and ensuring synthetic and biological feasibility. Experimental results validate the model’s robustness in structural modeling and its ability to generate functionally relevant peptides. Combined with physicochemical profiling and structural prediction, the framework enables controllable and biologically grounded peptide design for anticancer drug discovery.

Algorithm 2 outlines the diffusion-based ACP generation process. Biologically, the model learns the distribution of ACP feature representations through a diffusion-based generative process, and the generated peptides are subsequently organized in a cancer-type-aware manner to facilitate targeted downstream screening. After normalization and partitioning, a BFM-enhanced denoising network is trained to predict injected noise over sampled time steps using MSE loss. After training, DPM-Solver performs efficient reverse diffusion sampling to generate candidate peptide representations. The denoised features are decoded into peptides within a biologically plausible length range of 8–50 amino acids and exported for downstream evaluation, including physicochemical profiling, structure prediction, and simulation-based validation.

**Algorithm 2** Diffusion-based ACP Sequence Generation with Cancer-Type-Aware Organization

**Input:** ACP feature set 𝒳, cancer-type label *y*, feature dimension input_dim, total steps *T*, channels *C*, learning rate *η*, length range $\left[L_{\min},L_{\max}\right]$, number of samples *N*

**Output:** Trained model *M* and generated peptide sequences ${𝒮}_{y}$

1: **Initialization**

2: Encode cancer-type information c←LabelEncoder(y)   ▷Auxiliary semantic context for downstream organization

3: Initialize diffusion model M←initialize_diffusion_model(input_dim,T,C)

4: Compute diffusion coefficients β←Diffusion_Coefficients(T)

5: Define optimizer ←Adam(M,lr=η)

6: **Training loop**

7: **for** each training step **do**

8:  Sample real ACP feature x_start~𝒳

9:  Sample timestep t~Uniform(0,T)

10:  Sample noise ϵ~𝒩(0,I)

11:  Add noise: $x_{t}\leftarrow \text{q\_sample}(\beta ,x\_start,t)$

12:  Predict noise: $\hat{\epsilon }\leftarrow M(x_{t},t/T)$

13:  Compute loss: $L\leftarrow \text{MSE}(\hat{\epsilon },\epsilon )$

14:  Update parameters: $\theta \leftarrow \theta -\eta \nabla_{\theta }L$

15: **end for**

16: **Generation**

17: Initialize ${𝒮}_{y}\leftarrow \emptyset$

18: **for** *i* = 1 to *N* **do**

19:  Sample initial noise z~𝒩(0,I)

20:  Reverse diffusion (DPM-Solver): $z_{0}\leftarrow \text{dpm\_solver}(z,M,\beta )$

21:  Decode to peptide length $\left[L_{\min},L_{\max}\right]$: $seq\leftarrow \text{decode}(z_{0},[L_{\min},L_{\max}])$

22:   ${𝒮}_{y}\leftarrow {𝒮}_{y}\cup \{seq\}$

23: **end for**

24: Organize and export ${𝒮}_{y}$ according to cancer-type label *y*

25: **Return** $M,{𝒮}_{y}$

### 2.5 Evaluation indicators

The classification performance of the proposed model was evaluated using several standard metrics, including accuracy, precision, recall, F1-score, and the area under the receiver operating characteristic curve (AUC-ROC). These metrics provide a comprehensive assessment of model robustness, discriminative power, and generalization ability, particularly under imbalanced multi-class label distributions.

For the multi-class setting, precision, recall, and F1-score were computed on a per-class basis and then aggregated to obtain overall performance. This allows us to quantify how well each cancer-type-specific ACP class is recognized, while also summarizing the global behavior of the classifier. AUC-ROC was estimated in a one-vs-rest manner for each class and then averaged, reflecting the model’s ability to distinguish each ACP subtype from all others across all classification thresholds. Higher averaged AUC values indicate stronger overall discrimination among cancer types.

Recall characterizes the proportion of peptides from a given cancer-type class that are correctly identified, which is especially critical in imbalanced classification scenarios where certain cancer types are underrepresented. Precision measures the proportion of predicted samples for a specific class that truly belong to that class, indicating the reliability of the model’s predictions. The F1-score, defined as the harmonic mean of precision and recall, offers a balanced evaluation of sensitivity and specificity for each class and for the aggregated scores.

Although accuracy measures the overall proportion of correctly classified samples across all classes, it can be misleading in imbalanced multi-class datasets and should therefore be interpreted in conjunction with the other metrics. Taken together, these evaluation criteria provide a multidimensional and reliable framework for assessing model performance in multiclass anticancer peptide classification across diverse cancer types.

### 2.6 Biological and structural evaluation of generated peptides

To evaluate the biological relevance and structural feasibility of ACPs generated by the proposed conditional diffusion model, we conducted a comprehensive assessment that included sequence similarity, physicochemical properties, structural conformation, membrane interaction, and target-specific binding.

Homologous sequence alignment was performed using BLAST to compare the generated peptides with known functional protein fragments. BLAST alignment scores and E-values were examined to characterize local alignment patterns and residue-level similarities, with an emphasis on identifying conserved sequence motifs rather than establishing full-length homology. BLAST searches were conducted using a non-default E-value cutoff of $1\times 10^{-4}$, selected to balance sensitivity and specificity for short peptide sequences. Alignments with E-values above this cutoff were regarded as non-significant local matches. Key physicochemical parameters, including molecular weight, isoelectric point, instability index, aromaticity, and hydrophobicity, were computed using Biopython to assess solubility, structural stability, and membrane-binding potential. Structural flexibility was further evaluated using IUPred2A [55], which predicts disordered regions based on residue-level energy estimation. Representative sequences were modeled in three dimensions using AlphaFold2, selected for its wide adoption, established confidence metrics, and validated performance in peptide structure prediction. Given the short length and intrinsic flexibility of anticancer peptides, predicted structures with pLDDT values above 70 were considered indicative of reasonable local structural confidence, whereas very high pLDDT thresholds(e.g., > 90) are typically associated with rigid globular proteins. Predicted secondary structures were further annotated using DSSP to identify canonical structural elements such as *α*-helices and *β*-sheets. Together, these complementary analyses provide supporting evidence for the structural plausibility and biological feasibility of the generated ACPs.

**Fig 2:**
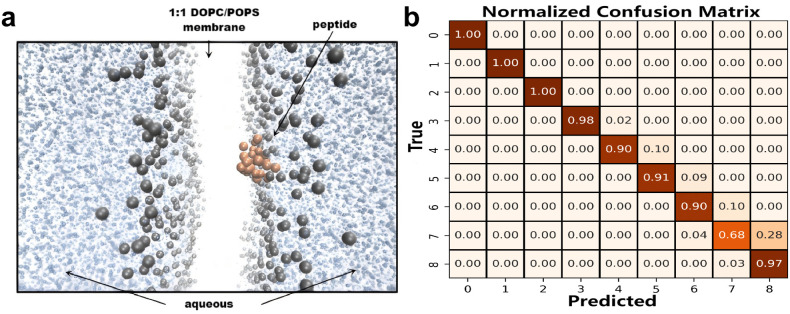
Characteristics of Anticancer Peptide–Membrane Interactions and Classification Performance with a Diffusion-Inspired Noise-Conditioned Classifier. **(a)** Coarse-grained molecular dynamics simulations demonstrate that anticancer peptides (orange) effectively bind to lipid bilayer membranes (gray), indicating significant interaction characteristics between the peptides and membranes. **(b)** The confusion matrix generated by the diffusion-inspired noise-conditioned classifier shows high classification accuracy for most categories, demonstrating that membrane interaction features provide crucial reference value for distinguishing anticancer peptide sequences.

To further investigate peptide interactions with cancer cell membranes and molecular targets, we performed molecular dynamics (MD) simulations and protein-protein docking analyses, as illustrated in Fig 2a. For membrane interaction analysis, three representative ACPs were modeled using AlphaFold V2. Full-atom MD simulations were conducted with GROMACS 2021.7 [56], following established protocols for peptide–membrane interactions [57,58]. Simulations used the CHARMM36 all-atom force field [59] and TIP3P water model under constant temperature and pressure. A membrane bilayer composed of DOPC and POPS lipids (1:1) was constructed, with peptides positioned above the membrane for energy minimization. Each system was simulated for 100 ns to evaluate peptide stability and interaction dynamics, based on RMSD, RMSF, and trajectory snapshots.

For target-specific binding analysis, the HER2 kinase domain (PDB ID: 7MN8) [60] was selected due to its clinical relevance in breast, gastric [61], and non-small cell lung cancers [62]. The structure was prepared by optimizing side-chain conformations and removing water molecules. Peptide–protein docking was performed using both HawkDock [63] and HDOCK [64], with binding regions defined according to the HER2 active site. Given the absence of experimentally resolved peptide–HER2 complex structures, docking analysis was conducted for qualitative assessment of interaction feasibility rather than quantitative affinity prediction. Docking reliability was assessed based on cross-method consistency between HawkDock and HDOCK, physical plausibility of binding conformations, and subsequent structural stability evaluated by molecular dynamics simulations, where the peptide RMSD relative to the initial docked conformation was monitored as a baseline indicator of pose consistency. Results from both platforms were compared to identify consistent binding poses, and docked complexes were visualized using PyMOL for structural interpretation.

## 3 Results

This section outlines the main experimental procedures, evaluation metrics, and data preprocessing methods used to evaluate the proposed model. Multiple approaches, including state-of-the-art methods, are compared to verify the effectiveness of the model’s classification module. In addition to traditional metrics like accuracy, recall, F1-score, and AUC-ROC, we also assess the model’s robustness under challenging conditions such as class imbalance and limited data scenarios. Specifically, the use of SMOTE-based oversampling and stochastic time-step conditioning is highlighted to demonstrate how these techniques address the challenges of data scarcity and class imbalance.

In parallel, biologically relevant indicators are employed to validate the sequences generated by the generative model, assessing both their functional reliability and biological adaptability. These include sequence similarity with known ACPs, physicochemical properties such as hydrophobicity and isoelectric point, and structural stability evaluated through AlphaFold2 and molecular dynamics simulations. The model’s ability to generate biologically plausible peptides is further validated through protein-protein docking and membrane interaction studies, specifically targeting HER2 kinase and other cancer-specific targets.

The diffusion-based generative mechanism is further evaluated to examine its ability to capture biologically meaningful peptide feature distributions. Cancer-type information is incorporated to organize and analyze the generated peptides, facilitating targeted screening toward biologically relevant regions of the feature space. As a result, the generated peptides exhibit both structural stability and functional relevance across different cancer types. In addition, we compare the proposed framework with traditional random sequence generators and discriminative classifiers, demonstrating its superior performance in both ACP classification accuracy and functional peptide generation.

### 3.1 Introduction to the dataset

**Fig 3:**
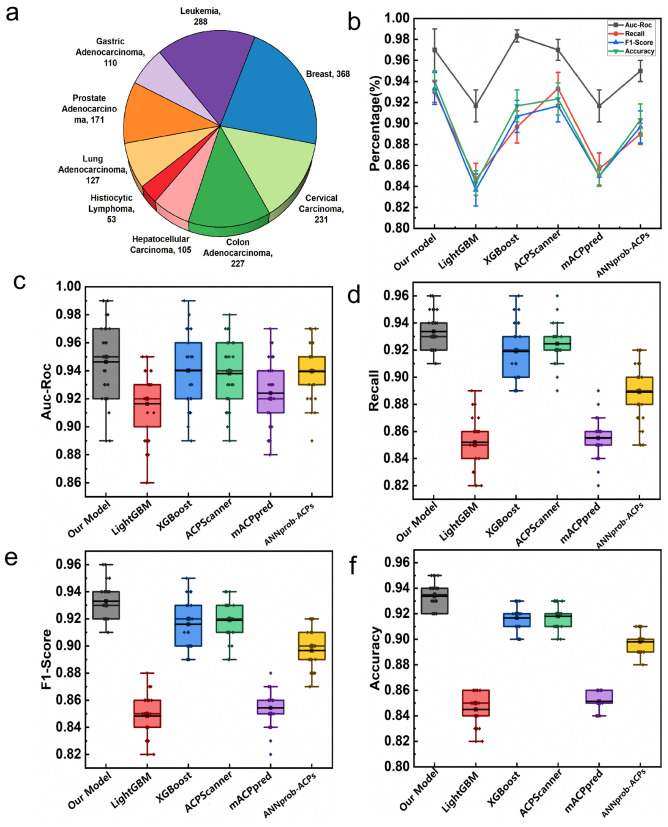
Performance comparison of different classification models across multiple evaluation metrics. **(a)** Pie chart showing the sample distribution of different anticancer peptide categories, with the number of samples labeled for each class. **(b)** Line chart comparing the average performance of different models across four evaluation metrics (AUC-ROC, recall, F1-score, and accuracy), with error bars representing standard deviation. **(c)** Box plots illustrating the distribution of AUC-ROC values for different models. **(d)** Box plots showing recall values for different cancer types. **(e)** Box plots displaying F1-score values for different models. **(f)** Box plots showing accuracy values for different models.

The dataset used in this study was curated from multiple publicly available ACP databases and relevant literature sources [65–68]. Each record contains a unique identifier, functional annotation, amino acid sequence, and the corresponding cancer type label. The functional summaries reported in Table 1 were derived from the functional annotations and mechanistic descriptions provided in the original database entries and associated literature, rather than from GO or KEGG enrichment analyses. For feature construction, each peptide sequence was encoded as a high-dimensional vector comprising amino acid composition, dipeptide frequency, and a comprehensive set of physicochemical descriptors. The sample distribution across different cancer types and the primary feature characteristics are summarized in Table 1 and illustrated in Fig 3a.

**Table 1:** Functional summary and sample size of anticancer peptides by cancer type.

Cancer Type	Functional Summary	Number	Label
Gastric Adenocarcinoma	Membrane disruption; anti-angiogenesis; invasion inhibition	110	0
Breast Adenocarcinoma	Anti-angiogenesis; pathway blockade; invasiveness reduction	368	1
Cervical Carcinoma	HPV targeting; apoptosis induction; immune activation	231	2
Colon Adenocarcinoma	Selective killing; anti-metastasis; low off-target toxicity	227	3
Hepatocellular Carcinoma	Proliferation suppression; immune clearance; invasion inhibition	105	4
Histiocytic Lymphoma	Apoptosis; membrane lysis; immune modulation	112	5
Leukemia	Anti-proliferation; apoptosis; immune stimulation	288	6
Lung Adenocarcinoma	Marker targeting; proliferation inhibition; toxicity control	127	7
Prostate Adenocarcinoma	Proliferation/metastasis inhibition; selective toxicity	171	8

### 3.2 Comparative evaluation of ACP prediction models

Experimental results demonstrate that the proposed diffusion-inspired, noise-conditioned classifier significantly outperforms existing methods in anticancer peptide (ACP) prediction, greatly improving classification accuracy and robustness. These findings underscore the model’s strong potential for practical applications in biomedical research and therapeutic peptide discovery.

The performance improvement is largely attributed to the model’s unique architecture, particularly the multiscale feature interaction module and the diffusion-inspired noise-conditioning strategy. The multiscale feature interaction module plays a vital role in capturing both local structural features and global sequence dependencies, which are crucial for accurate classification. By processing features at multiple resolutions, the module enables the model to distinguish fine-grained sequence patterns from broader structural features, significantly enhancing its generalization ability. Meanwhile, the noise-conditioning mechanism injects a stochastic time-dependent signal into the fused representations, allowing the classifier to learn smoother and more robust decision surfaces that better capture the multimodal structure of peptide data. The inclusion of cancer-type-specific label embeddings further refines this process, ensuring that the learned representations align with biologically relevant features and improving the detection of minority classes where traditional discriminative models often struggle.

As shown in Fig 3c–3f, the model demonstrates stable performance across key metrics (accuracy, AUC-ROC, recall, and F1-score), with minimal variance and few outliers. This indicates consistent performance across validation folds and subsets. In Fig 3b, a comparative evaluation illustrates that the proposed model consistently outperforms several state-of-the-art methods, including ACPScanner, mACPpred, ANNprob-ACPs, XGBoost [69], and LightGBM [70], across all metrics. Specifically, the model achieves an AUC-ROC of 0.99, recall of 0.93, F1-score of 0.93, and accuracy of 0.94, all superior to existing ACP classifiers, as detailed in Table 2. The low standard deviations further validate the model’s stability.

**Table 2:** Performance comparison across models.

Model	AUC-ROC	Recall	F1-Score	Accuracy
Our Model	**0.99 ± 0.01**	**0.93 ± 0.01**	**0.93 ± 0.01**	**0.94 ± 0.01**
LightGBM	0.91 ± 0.04	0.84 ± 0.03	0.83 ± 0.04	0.83 ± 0.03
XGBoost	0.98 ± 0.01	0.91 ± 0.02	0.90 ± 0.02	0.91 ± 0.02
ACPScanner	0.97 ± 0.02	0.93 ± 0.02	0.92 ± 0.02	0.93 ± 0.02
mACPpred	0.91 ± 0.03	0.89 ± 0.03	0.88 ± 0.03	0.89 ± 0.02
ANNprob-ACPs	0.94 ± 0.02	0.88 ± 0.03	0.87 ± 0.02	0.88 ± 0.02

Although some baseline models, like the multi-layer perceptron (MLP), show competitive performance on individual metrics such as AUC, they lack consistency across multiple evaluation criteria, indicating limited generalizability. In contrast, the proposed model exhibits strong performance across all metrics, confirming its robustness. The normalized confusion matrix in Fig 2b further validates the classification reliability. Most diagonal elements approach 1.0, indicating high accuracy across all nine classes. The model effectively minimizes false positives and false negatives, even in challenging class boundaries, such as between labels 6 and 7.

To alleviate class imbalance, we combine a SMOTE-based oversampling strategy with a class-balanced training objective. During training, synthetic samples are generated for minority classes using SMOTE, and a weighted cross-entropy loss with class weights inversely proportional to class frequencies is applied. Under highly imbalanced class-frequency distributions (e.g., majority-to-minority ratios exceeding 4:1), the model still maintains stable performance without resorting to more elaborate imbalance-handling techniques (such as focal loss, two-stage resampling, or advanced cost-sensitive schemes). This suggests that the diffusion-inspired noise-conditioning mechanism provides strong generative-style regularization, preserving minority-class characteristics by modeling the underlying data distribution rather than merely learning decision boundaries.

In conclusion, the proposed method offers superior performance in multiclass ACP classification by combining architectural innovation with enriched feature representations. Compared to existing models and mainstream classifiers, it demonstrates stronger generalization under complex structural variations and class-imbalanced settings, providing a reliable, scalable, and biologically meaningful framework for functional peptide identification.

### 3.3 Feature space dimensionality reduction and clustering visualization

To address the class imbalance in the training set, the SMOTE was applied. SMOTE generates synthetic samples for the minority class by interpolating between existing instances, thus expanding the dataset and enhancing its diversity. This oversampling technique effectively mitigates the challenges posed by underrepresented categories, significantly improving the model’s ability to generalize, particularly under conditions of data scarcity and class imbalance.

The fused feature space, originally comprising 1,454 dimensions, was reduced to 200 dimensions using Principal Component Analysis (PCA). This dimensionality reduction not only preserves over 95% of the original variance but also significantly reduces computational cost, accelerating the training process without compromising classification accuracy. The combination of SMOTE-based oversampling and PCA ensures that the model can maintain high accuracy and efficiency, even with a limited number of training samples.

**Fig 4:**
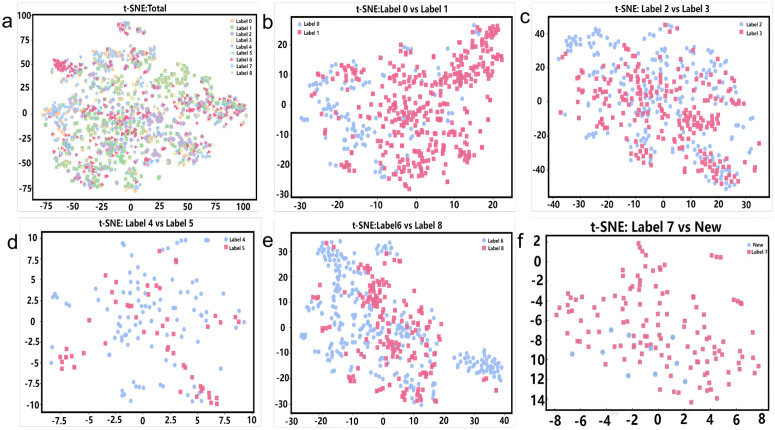
t-SNE visualizations of feature representations for different label combinations. (a) t-SNE projection of the original feature space across all peptide categories, showing the overall distribution of multiple labels. (b) t-SNE visualization of feature representations for Label 0 and Label 1. (c) t-SNE visualization of feature representations for Label 2 and Label 3. (d) t-SNE visualization of feature representations for Label 4 and Label 5. (e) t-SNE visualization of feature representations for Label 6 and Label 8. (f) t-SNE projection of feature representations for Label 7 and newly generated anti-lung cancer peptide samples.

To assess the discriminative capability of the learned features, t-distributed Stochastic Neighbor Embedding (t-SNE) was employed for visualization. As shown in Fig 4a-4e, peptide categories form well-defined clusters in the reduced latent space, indicating strong inter-class separability. In two-class comparisons, categories with distinct structural or physicochemical properties exhibit clear separation, while biologically similar categories show partial overlap. Notably, the overlap between known lung cancer peptides and generated candidates suggests that the model effectively captures biologically relevant sequence features, facilitating the generation of functionally relevant peptides.

By combining SMOTE-based oversampling with dimensionality reduction, the framework not only enhances feature quality but also successfully mitigates the impact of class imbalance and data scarcity. This dual approach significantly improves the model’s ability to generalize, offering a robust solution for ACP classification under challenging dataset conditions, particularly when dealing with sparse data and imbalanced classes. The model’s stability and accuracy in these scenarios make it well-suited for real-world applications in peptide classification and cancer therapy development.

### 3.4 Prediction and validation analysis of newly discovered anticancer peptides

To evaluate the generalization capability of the proposed model on unseen data, we collected ten recently reported anti-lung cancer peptide sequences that were not included in the training dataset from public databases and literature, as summarized in Table 3. This functional category was selected to test the model’s performance in a small-sample, single-class scenario.

**Table 3:** Sequences and validation results of 10 newly discovered anti lung cancer peptides predicted by the models.

ID	Name	Sequence	Confirm
DBAASPR_21170	Brevinnin 1 AW	FLPLGLAANFLPQIC KIARKC	Yes
DBAASPR_18209	Temporin HLa, Temporin FL	FPFLFGIALSSLPKIL	Yes
DBAASPR_15994	Kassinatuerin 3	FIQHILPLPIHAIQGKIDIF	Yes
DBAASPR_15602	Brevinnin 1Ghd, Brevinnin 1HL	FLGALFKVASKLVPA AICSISKC	Yes
DBAASPR_13836	Tat (48–56) + PPF BBI [5 –15]	RKKRQRRRRCWTKS IPPKPC	Yes
DBAASPR_13833	PPF BBI [P16]	ALRGCWTKSIPPKC	Yes
DBAASPR_13832	PPF BBI [K8F]	ALRGCWTKSIFPPC	Yes
DBAASPR_13831	PPF BBI	ALRGCWTKSIPPKC	Yes
DBAASPR_12644	Japonicin 2LF	FIVPSIFLLKAFICA LKCC	Yes
DBAASPS_887	Buforin 2 (5–13) [17–203]	RAGLQFVGGRRLLR RRRRRRR	No

The model correctly identified nine out of ten sequences as anticancer peptides, achieving a prediction accuracy of 90%. These results highlight the robustness and practical applicability of the model for recognizing previously unseen peptides, even with limited data and restricted category coverage.

Feature-level discrimination was further assessed through principal component analysis (PCA) and t-distributed stochastic neighbor embedding (t-SNE) visualizations. As shown in Fig 4f, PCA demonstrated that the model captures dominant sequence features in high-dimensional space, while t-SNE projections revealed compact intraclass clustering and clear interclass separation in the reduced-dimensional space.

Although the independent test set contained only a single functional class, the model maintained stable and accurate predictions, confirming its scalability and real-world applicability for functional peptide identification.

### 3.5 Functional and structural evaluation of generated peptides

To evaluate the model’s adaptability and generation quality across different functional categories and sequence lengths, we selected three representative labels from the nine ACP classes, namely label 0 (structurally stable peptides), label 1 (structurally heterogeneous peptides), and label 7 (functionally diverse peptides). This selection spans a broad range of biological complexity and provides a representative basis for assessing model generalization.

For each label, the model generated peptide sequences at predefined lengths, from which candidates with high classification confidence and plausible physicochemical properties were selected for further analysis. BLAST local alignments against known ACPs in the DCTPep database [71] were performed to examine residue-level similarities and conserved sequence patterns. As summarized in Table 4, the selected peptides exhibit reasonable alignment coverage and conserved local similarities, supporting their functional relevance while avoiding near-duplicate matches to the training data.

**Table 4:** BLAST local alignment summary for three representative generated peptides (INY23, KFS19, and KVY25) against the training dataset and the DCTPep database. The table reports the best local alignments, including alignment score, E-value, coverage, identity, positive similarity, and gap percentage, to illustrate conserved residue-level similarities supporting peptide selection. Given the short length of ACPs, BLAST results are interpreted at the level of local alignment rather than full-length homology.

Peptide	Database	Score	E-value	Coverage (%)	Identity (%)	Positive (%)	Gaps (%)
INY23	Training set	16.3	6.2	46.2	42	75	8
INY23	DCTPep	16.3	6.2	46.2	42	75	8
KFS19	Training set	16.2	4.4	31.8	71	71	12
KFS19	DCTPep	16.2	4.4	31.8	71	71	12
KVY25	Training set	21.4	1.6	96.4	86	100	12
KVY25	DCTPep	21.4	1.6	96.4	86	100	12

**Fig 5:**
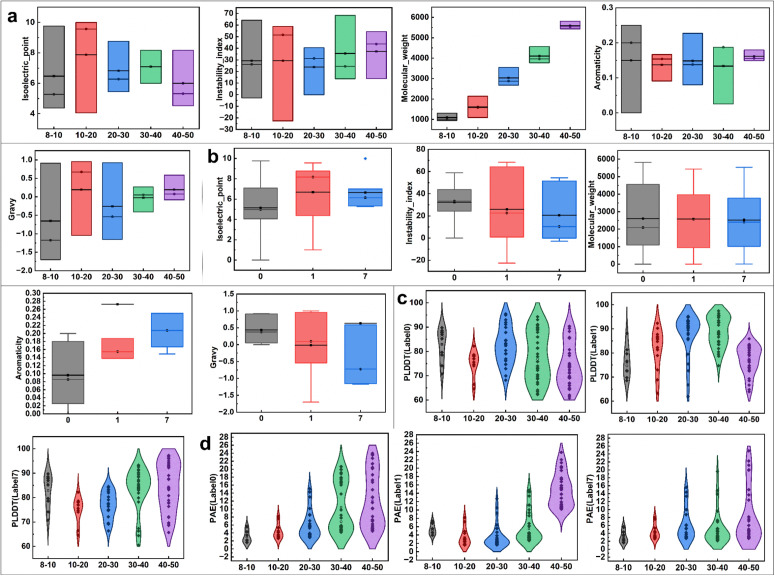
Visualization of the distributions of the biological properties and structural characteristics of anticancer peptide samples under different features and structural states. **(a)** Box plots showing the distributions of physicochemical properties, including molecular weight, instability index, isoelectric point, aromaticity, and hydrophobicity, for anticancer peptides with different sequence lengths. **(b)** Box plots showing the distributions of physicochemical properties for anticancer peptides with different labels. **(c)** Violin plots showing the distributions of pLDDT values for anticancer peptides with different labels and lengths, indicating the model reliability. **(d)** Violin plots showing the distributions of PAE values for anticancer peptides with different labels and lengths, indicating the structural prediction spatial accuracy.

Physicochemical properties varied consistently across the selected labels. The activity score reported in Table 5 represents a relative model-derived prediction metric rather than an experimentally calibrated biological activity, and no fixed threshold was applied to define low, moderate, or high activity levels. As shown in Fig 5a, 5b and summarized in Table 5, molecular weight scaled with peptide length. Isoelectric points displayed label-associated trends, with peptides in label 1 generally exhibiting higher basicity and those in label 0 tending toward acidic profiles. Instability indices were more variable in label 1, indicating increased structural flexibility. Aromaticity showed a moderate increase in label 7, whereas hydrophobicity remained relatively stable across all labels. These observations indicate that the generated peptides exhibit coherent physicochemical characteristics across different functional categories.

**Table 5:** Physicochemical properties and bioactivity scores of 15 artificial anticancer peptide sequences designed by AI.

Sequence	Length	Charge	H	*μ*H	Activity Score
ITFIQFRMIH	10	1	0.91	0.27	0.47
YVSPAEASLVG	11	-1.09	0.67	0.09	0.23
INYQKARGVKSQNVINQNRVTIAG	26	3.91	-0.44	0.26	0.92
HTIRLMTQPQLEHPTQLVMWQTHTVKCLEVAMHVVARID	39	0.23	0.05	0.32	0.72
VSNGVRDDYWTYDDVAYSYHAENIVATQEWKNTFVLIMSRAFSSAFLCI	50	-3.05	0.08	0.05	0.323
LQENDRAT	8	-1.09	-1.7	0.45	0.88
PTARVIVWVYRCI	13	1.86	0.95	0.59	0.94
KFSMDFFSCEWIPSTCRANNS	22	-0.18	-0.2	0.27	0.93
FLAHIVAFIQNLQNMGKRWYEYRCMELPVLYI	32	0.95	0.27	0.21	0.3
DQWGPIKPQYHFIAQPV MKWYLIFQLHCIGDHTINIYSHSVRL	45	1.22	-0.08	0.16	0.54
MYQTNFNP	8	-0.09	-1.18	0.19	0.35
AFNKYRVATQHQNWSPGM	18	2	-1.04	0.12	0.38
KVYSSDAWGSMIMNCGEWKLFKVQKWCM	28	1.81	-0.17	0.09	0.79
HWIWSKCQNNISGEFIMTFDFGVVFGQYSKPH	32	-0.96	-0.41	0.09	0.80
DMHMGIDGHWCSLFSWLFCENINRSVTIIMLFWLGAVRVNSVAMEI	47	-2	0.59	0.15	0.72

Disorder predictions using IUPred2A, reported in Table 6, revealed that peptides from label 0 were predominantly ordered. In contrast, label 1 sequences exhibited higher disorder, while label 7 peptides displayed a mixture of ordered and disordered regions, reflecting diverse functional properties. In this analysis, residues with disorder scores exceeding 0.5 were interpreted as having a high disorder propensity, indicating reduced structural ordering or increased flexibility rather than global structural instability.

**Table 6:** Structural disorder score and conclusion categorization of different label generated peptides.

Label	Protein sequence	Length	Average score	Maximum score	Number of residuals exceeding the threshold (0.5)	Conclusion
0	ITFIQFRMIH	10	0.002	0.002	0	Highly organized structure
0	YVSPAEASLVG	11	0.101	0.101	0	Medium structural ordering
0	INYQKARGVKSQNVINQNRVTIAG	26	0.006	0.011	0	Highly organized structure
0	HTIRLMTQPQLEHPTQLVMWQTHTVKCLEVAMHVVARID	39	0.252	0.48	0	Medium structural ordering
0	VSNGVRDDYWTYDDVAYSYHAENIVATQEWKNTFVLIMSRAFSSAFLCI	50	0.031	0.073	0	Highly organized structure
1	LQENDRAT	8	0.976	0.976	8	High degree of disorder
1	PTARVIVWVYRCI	13	0.005	0.006	0	Highly organized structure
1	KFSMDFFSCEWIPSTCRANNS	22	0.2	0.349	0	Highly organized structure
1	FLAHIVAFIQNLQNMGKRWYEYRCMELPVLYI	32	0.005	0.011	0	Highly organized structure
1	DQWGPIKPQYHFIAQPV	16	0.027	0.027	0	Highly organized structure
1	MKWYLIFQLHCIGDHTINIYSHRVL	45	0.027	0.167	0	Highly organized structure
7	MYQTNFNP	8	0.637	0.637	8	High degree of disorder
7	AFNKYRVATQQHNWSPGM	18	0.457	0.637	4	Significant localized disorder
7	KVYSSDAWGSMIMNCGEWKLFKVQKWCM	28	0.157	0.254	0	Medium structural ordering
7	HWIWSKCQNNISGEFIMTFDFGVVFGQYSKPH	32	0.101	0.176	0	Medium structural ordering
7	DMHMGIDGHWCSLFSWLFCENINRSVTIIMLFWLGAVRVNSVAMEI	47	0.007	0.017	0	Highly organized structure

To further assess structural reliability, fifteen representative peptides were modeled using AlphaFold2. Most predicted structures achieved pLDDT scores above 70, consistent with the confidence criterion defined for short and flexible peptides, and exhibited compact predicted aligned error distributions, as shown in Fig 5c, 5d. The predicted secondary structures were primarily composed of *α*-helices, with moderate occurrences of *β*-sheets and random coils, demonstrating structural diversity and biological plausibility.

Overall, these results demonstrate the framework’s ability to generate structurally stable and biologically plausible peptide candidates across diverse functional categories, supporting its applicability to rational ACP design.

### 3.6 Structural stability and membrane interaction analysis of selected peptides

To identify peptide molecules with potential anticancer activity, sequences from the DCTPep database were filtered using BLAST homology search and activity scores from PeptideRanker [72]. Sequences with scores above 0.8 were prioritized for downstream validation.

Three representative candidates (INY23, KFS19, and KVY25) were selected for structural modeling and membrane interaction analysis. Their 3D structures were predicted using AlphaFold V2, followed by molecular dynamics simulations to evaluate conformational stability and membrane affinity.

**Fig 6:**
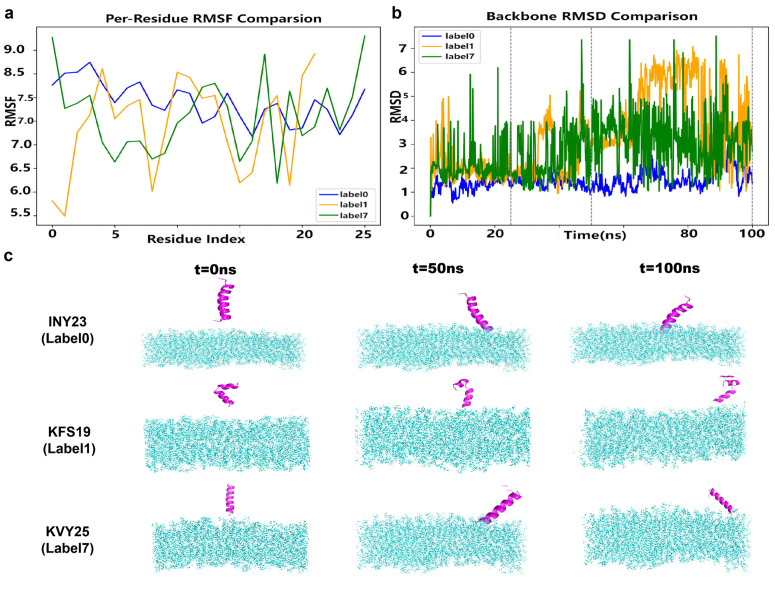
[MD simulation of peptide–membrane interactions] Molecular dynamics simulation analysis of the interactions between representative generated peptides and lipid membranes. **(a)** Per-residue root mean square fluctuation (RMSF) profiles of peptides with different labels, illustrating local residue flexibility during the simulations. **(b)** Backbone RMSD (C*α*) trajectories of peptides over the simulation time, calculated relative to the initial structures, with gray dashed lines indicating selected time points used for structural comparison. **(c)** Representative snapshots of peptide–membrane systems at 0, 50, and 100 ns for peptides INY23, KFS19, and KVY25, showing conformational evolution and membrane-association behavior over time. The RMSD and RMSF metrics are used to assess conformational stability and membrane interaction dynamics, rather than to infer biological activity or docking accuracy.

As shown in Fig 6, INY23 exhibited the lowest RMSF, as illustrated in Fig 6a, and demonstrated the most stable RMSD trajectory, as presented in Fig 6b. It maintained a consistent vertical insertion into the membrane throughout the simulation, as shown in Fig 6c. KVY25 displayed moderate structural stability and maintained membrane contact but lacked a firm transmembrane conformation. In contrast, KFS19 exhibited significant fluctuations in both RMSF and RMSD, gradually detaching from the membrane and undergoing conformational collapse.

Overall, INY23 demonstrated superior structural stability and membrane-association behavior under the simulated conditions, supporting its prioritization as a promising candidate for further investigation. The comparative results provide a biophysical perspective on peptide–membrane interactions and illustrate how conformational stability and membrane interaction patterns can serve as informative indicators in computational ACP screening.

Importantly, these observations should be interpreted as biophysical consistency rather than direct evidence of anticancer activity. The superior behavior of INY23 can be attributed to its balanced hydrophobic–hydrophilic profile and helical stability, highlighting the importance of sequence-level design constraints in shaping peptide–membrane interactions. Notably, the agreement between sequence-level predictive scoring (PeptideRanker) and subsequent structural and dynamical assessments reflects the internal coherence of the proposed screening pipeline across different evaluation layers. Together, this integrated strategy demonstrates how generative modeling combined with multiscale biophysical analysis can support rational prioritization of ACP candidates for downstream experimental validation.

### 3.7 HER2-targeted binding analysis of designed anticancer peptides

**Fig 7:**
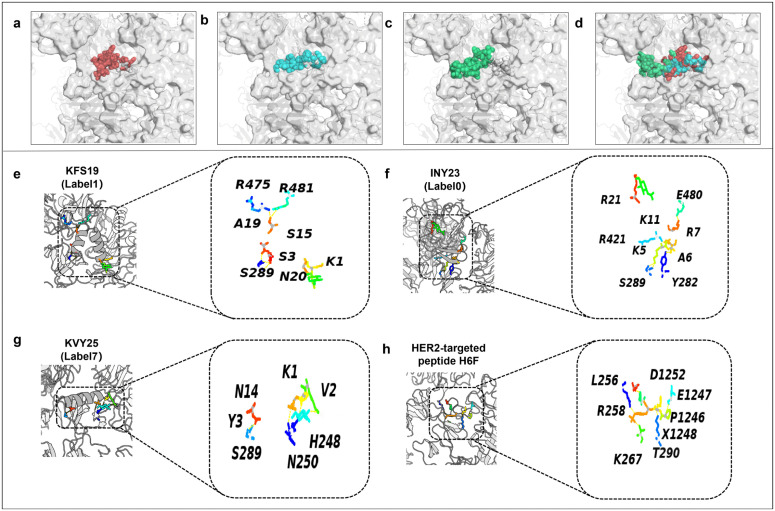
Predicted docking conformations and interaction patterns of representative peptides with the HER2 kinase domain. **(a–c)** Predicted docking poses of KFS19, INY23, and KVY25 within the HER2 catalytic region. **(d)** Superimposed docking poses of the three generated peptides together with the control peptide H6F, illustrating their relative spatial distributions and partial overlap within the binding region. **(e–g)** Close-up views of residue-level interaction patterns for KFS19, INY23, and KVY25, highlighting residues involved in predicted polar and hydrogen-bond interactions. **(h)** Predicted docking pose and interaction pattern of the HER2-targeted control peptide H6F. Docking results are presented for qualitative structural interpretation and comparative analysis of peptide–HER2 interaction feasibility. Reported docking poses and interaction patterns should not be interpreted as quantitative binding models.

In this study, anticancer peptides KFS19 (breast cancer), INY23 (gastric cancer), KVY25 (lung cancer), and the HER2-targeted control peptide H6F [73], a synthetic peptide previously validated in wet-lab studies for HER2 targeting, were docked to the HER2 kinase domain (PDB ID: 7MN8) to explore potential peptide–target interaction patterns. The docking results are illustrated in Fig 7, with detailed docking scores and confidence values summarized in Table 7.

**Table 7:** Docking scores and confidence values of anticancer peptides against HER2.

Ligand Protein (Binding Compound)	Confidence Score	Docking Score	Docking Energy Score (kcal/mol)
Breast cancer KFS19 (Label1)	0.8675	−243.97	−43.67 (kcal/mol)
Gastric cancer INY23 (Label0)	0.8154	−224.28	−33.09 (kcal/mol)
Lung cancer KVY25 (Label7)	0.8137	−223.71	−30.54 (kcal/mol)
HER2-targeted peptide H6F	0.5212	−154.24	−23.59 (kcal/mol)

Among the peptides, KFS19 exhibited the most favorable predicted docking score and confidence value among the tested candidates, forming multiple polar contacts with HER2 residues such as R475, R481, and S289. INY23 and KVY25 also produced consistent docking poses with relatively favorable scores, engaging residues including S289, E480, and N250 that are located within or proximal to the HER2 active region. Although the reported docking energies should not be interpreted as quantitative binding free energies, the relative ranking of docking scores suggests that these peptides share comparable interaction propensities and partially overlapping binding regions on HER2.

In contrast, the control peptide H6F showed less favorable docking scores and confidence values, with predicted binding poses deviating from the core catalytic pocket. The reduced spatial overlap and fewer stabilizing contacts suggest a lower likelihood of stable association under the docking conditions.

Overall, the docking analysis provides a qualitative and comparative perspective on peptide–HER2 interaction feasibility. The observed consistency in predicted binding regions among the generated peptides, together with their relative docking preferences compared to the control peptide, supports their prioritization for further structural and biophysical investigation rather than constituting direct evidence of binding affinity or therapeutic efficacy.

### 3.8 Motif-and structure-level comparison with known anticancer peptides

In addition to molecular dynamics simulations, we performed motif- and structure-level comparisons between the generated peptides and known anticancer peptides to evaluate whether the model captures biologically relevant features beyond dynamic stability.

Representative known anticancer peptides were selected from the original dataset as reference controls, all of which possess experimentally or literature-supported anticancer activity and were not generated by the model.

**Fig 8:**
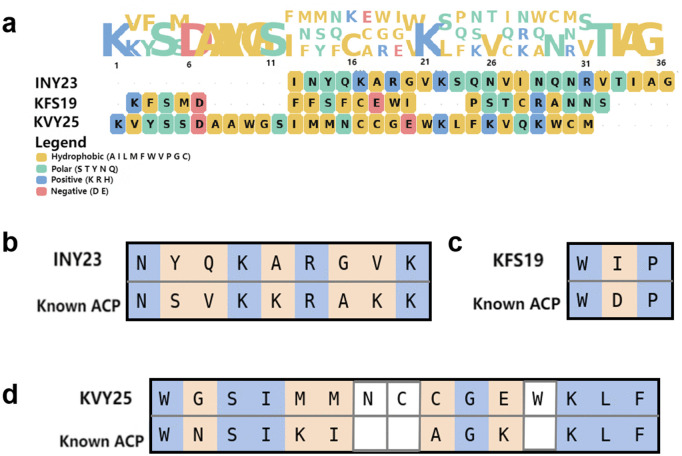
Motif- and sequence-level comparison between generated peptides and known anticancer peptides. **(a)** Sequence motif distributions of the generated peptides, showing enrichment of hydrophobic and positively charged residues. **(b)** Local Smith–Waterman alignment between INY23 and a reference peptide from the original dataset, revealing limited residue-level similarity. **(c)** Short local alignment of KFS19, indicating minimal sequence overlap with known peptides. **(d)** Local alignment of KVY25, where conserved residues are interspersed with mismatches and gaps, indicating the absence of global sequence homology.

Sequence motif visualization shows that the generated peptides display characteristic distributions of positively charged (K/R), hydrophobic, and aromatic residues commonly observed in anticancer peptides, as illustrated in Fig 8a and summarized in Table 8. The enrichment of basic residues and the frequent proximity between aromatic and basic residues are consistent with electrostatically driven membrane interactions.

**Table 8:** Motif-based functional characterization of generated ACPs.

Peptide	Length	Basic Residues (K/R)	Aromatic Residues (W/F/Y)	Aromatic–Basic Proximity	Amphipathic Evidence
INY23	24	4 (K2, R2)	1 (Y)	Present	Helical-prone
KFS19	20	1 (K)	3 (F2, W1)	Present	Moderate
KVY25	27	4 (K3, R1)	3 (W2, F1)	Strong	Helical-prone

**Fig 9:**
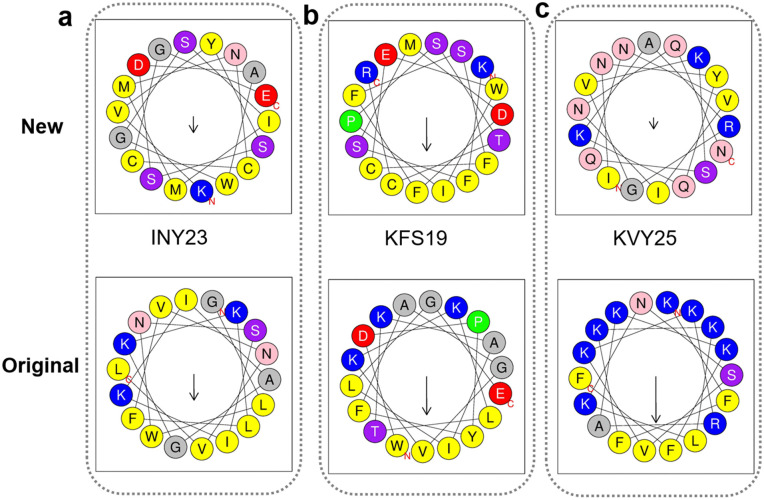
Amphipathic helical motif comparison between generated and known anticancer peptides. **(a)** Helical wheel projection of the generated peptide INY23, illustrating its amphipathic *α*-helical organization. **(b)** Helical wheel projection of the generated peptide KFS19, showing the spatial segregation of hydrophobic and positively charged residues. **(c)** Helical wheel projection of the generated peptide KVY25, illustrating an amphipathic helical arrangement.

To assess sequence-level similarity, local Smith–Waterman [74] alignments were conducted between the generated peptides and their corresponding reference peptides from the original dataset. As shown in Fig 8a–8d, only short locally aligned segments with limited residue-level similarity were identified, indicating the absence of global sequence homology and excluding direct sequence memorization.

At the structural motif level, helical wheel [75] projections demonstrate that both the generated peptides and the reference anticancer peptides adopt amphipathic *α*-helical organizations, characterized by spatial segregation of hydrophobic and positively charged residues on opposite faces, as shown in Fig 9a–9c. Such amphipathic architectures are well-established structural determinants for membrane binding and disruption.

Collectively, these motif- and structure-level analyses indicate that the generative model captures key functional and stru ctural characteristics of anticancer peptides while avoiding direct reproduction of sequences from the original dataset.

## 4 Discussion

This study presents a fusion-driven unified framework for anticancer peptide (ACP) discovery that integrates diffusion-inspired classification and diffusion-based sequence generation within a shared feature space. The architecture addresses key challenges in ACP modeling, including class imbalance, sequence heterogeneity, and the integration of predictive and generative objectives within a biologically coherent framework.

From a predictive perspective, integrating ProtBERT-derived embeddings with physicochemical descriptors through the Multiscale Embedding Compression Strategy (MECS) enables complementary representation learning. The multiscale attention mechanism enhances biologically informative features while suppressing redundancy, contributing to improved robustness under imbalanced multi-class settings. The diffusion-inspired noise-conditioning mechanism further regularizes the feature space by encouraging smoother decision boundaries and reducing overfitting.

Compared with purely cost-sensitive strategies, SMOTE-based oversampling operates in feature space and preserves label-conditioned structural distributions, making it compatible with the proposed encoder. By expanding minority-class manifolds prior to noise perturbation, the framework improves representation stability without compromising class separability.

In the generative component, the denoising diffusion framework with BFM and TFAM enables temporal–semantic refinement during reverse diffusion. Compared with GAN- or VAE-based approaches, diffusion modeling provides stable training and consistent exploration of sequence space. Cancer-type-aware conditioning further constrains generation within biologically meaningful subspaces rather than unconstrained sampling.

Structural modeling, molecular dynamics simulations, and docking analyses suggest that the generated peptides exhibit plausible conformational stability and membrane interaction behavior. Docking results were interpreted qualitatively as structural feasibility assessments rather than quantitative binding predictions.

To mitigate data leakage risk, highly similar sequences were removed during preprocessing, and alignment analyses confirmed the absence of global memorization. These observations indicate that the model captures functional sequence patterns rather than reproducing training fragments.

Several limitations remain. Extremely rare peptide subclasses may benefit from advanced rebalancing strategies such as few-shot or meta-learning approaches. The tendency toward *α*-helical conformations likely reflects dataset bias and the prevalence of membrane-active ACPs. Incorporating structure-aware regularization may promote greater conformational diversity. Finally, experimental validation is required to confirm biological activity and safety.

Overall, the proposed framework demonstrates how fusion-driven diffusion modeling can unify predictive discrimination and controlled sequence generation within an interpretable architecture, contributing to ACP discovery and broader data-efficient biomolecular modeling efforts.

## 5 Conclusion

In summary, we developed UACD-ACPs, a fusion-guided and diffusion-enhanced framework for anticancer peptide classification and generation. By integrating multilevel feature fusion with diffusion-inspired noise conditioning and attention-enhanced generative refinement, the model achieves robust multiclass ACP prediction while producing structurally coherent and biologically plausible peptide candidates. The unified architecture provides a scalable and biologically grounded computational strategy for peptide-based therapeutic discovery.

Beyond anticancer peptides, the modular fusion–diffusion paradigm introduced in this study may be extended to other functional peptide design tasks, protein engineering applications, and precision medicine contexts. This work highlights the potential of integrating advanced representation learning with biologically informed validation to accelerate rational therapeutic peptide development.
